# Three‐month outcomes of aortic valve reconstruction using collagenous membranes (biosheets) produced by in‐body tissue architecture in a goat model: a preliminary study

**DOI:** 10.1186/s12872-021-01988-6

**Published:** 2021-04-15

**Authors:** Keitaro Okamoto, Tadashi Umeno, Takashi Shuto, Tomoyuki Wada, Hirofumi Anai, Haruto Nishida, Yasuhide Nakayama, Shinji Miyamoto

**Affiliations:** 1 Department of Cardiovascular Surgery, Faculty of Medicine, Oita University, 1-1 Idaigaoka, Yufu , Oita 879-5593 Japan; 2 Department of Cardiovascular Surgery, Oita University Hospital, Oita, Japan; 3 Clinical Engineering, Faculty of Medicine, Oita University, Yufu, Oita Japan; 4 Department of Pathology, Oita University Hospital, Oita, Japan; 5 Biotube Co., Ltd, Tokyo, Japan

**Keywords:** Aortic root, Valve repair, Tissue Engineering, Valve replacement

## Abstract

**Background:**

Autologous pericardium is widely used as a plastic material in intracardiac structures, in the pulmonary artery, and in aortic valve leaflets. For aortic valve reconstruction (AVRec) using the Ozaki procedure, it has produced excellent clinical results over a 10-year period. In-body tissue architecture (iBTA), which is based on the phenomenon of tissue encapsulation of foreign materials, can be used to prepare autologous prosthetic tissues. In this preliminary study, we examined whether biosheets can be used as valve leaflet material for glutaraldehyde-free AVRec by subchronic implantation experiments in goats and evaluated its performance compared with glutaraldehyde-treated autologous pericardium for AVRec.

**Methods:**

Biosheets were prepared by embedding molds for two months into the dorsal subcutaneous spaces of goats. Autogenic biosheets (n = 4) cut into the shape of the valve were then implanted to the aortic valve annulus of four goats for three months without glutaraldehyde treatment. Autologous pericardium (n = 4) was used in four goats as a control. Valve function was observed using echocardiography.

**Results:**

All goats survived the three-month study period. With biosheets, the leaflet surfaces were very smooth and, on histology, partially covered with a thin neointima (including endothelial cells). Biosheets were more thoroughly assimilated into the aortic root compared with autologous pericardium.

**Conclusions:**

For the first time, biosheets were used for large animal AVRec. Biosheets could function as leaflets in the aortic position and may have the ability to assimilate into native valves.

## Background

Valvular dysfunction due to heart disease requires frequent reconstruction using mechanical valves, biological valves, or autologous pericardium valves. Autologous pericardium is widely used as a plastic material in intracardiac structures, in the pulmonary artery, and in aortic valve leaflets. For aortic valve reconstruction (AVRec) using the Ozaki procedure, autologous pericardium is used after fixation with glutaraldehyde. This has produced excellent clinical results over a 10-year period [1]. This method has almost no immunologic reaction, possessing the characteristics of its natural counterparts. However, fixation with glutaraldehyde is required for this method. Glutaraldehyde fixation limits growth adaptability. Tissue engineering applies methods of scientific engineering to create viable structures. In-body tissue architecture (iBTA), which is based on the phenomenon of tissue encapsulation of foreign materials, can be used to prepare autologous prosthetic tissues [2]. This technology has been successfully applied to the engineering of cardiovascular tissues, of vascular grafts as biotubes, or of heart valve-like tissues as biovalves. Biosheets, which are iBTA-induced membranous tissues, have been applied to the cornea [3], as well as to the repair of the diaphragm [4], esophagus [5], trachea [6] and blood vessels [7, 8]. The studies that have reported on this technology indicated the possibility of tissue regeneration, self-repair, and growth adaptability. This technology could have potential for use as an alternative aortic leaflet material in AVRec.

In this study, we examined whether biosheets can be used as aortic valve leaflet material for glutaraldehyde-free AVRec by subchronic implantation experiments using goats and evaluated its performance and histology compared with glutaraldehyde-treated autologous pericardium for original AVRec.

## Methods

### **Animal procedures**

Eight adult Saanen goats (body weight, 51.1 ± 6.4 kg) were purchased from Inoue Farm (Gunma, Japan). An approval/permission from the farm owner to use the animals was not necessary. Animals were housed one in each cage under the same conditions, with dark-light cycles of 12 h and constant temperature of 24 ± 2 °C with ad libitum access to food and water. The animals had olfactory, visual, and auditory contact with the other goats. Animals were distributed randomly into different groups. All animals were cared for in accordance with the “Guide for the Care and Use of Laboratory Animals”, published by the US National Institutes of Health (NIH publication No. 85 − 23, revised 1996). Oita University Animal Ethics Committee approved the protocols (OITA-R022001) used in the present study. AVRec was performed in eight goats using different materials: biosheet or glutaraldehyde-fixed autologous pericardium. Animals were sacrificed by intravenous injection of KCL three months after AVRec and the hearts were harvested as samples for morphological observation.

### Preparation of Autogenic biosheets

**Fig. 1:**
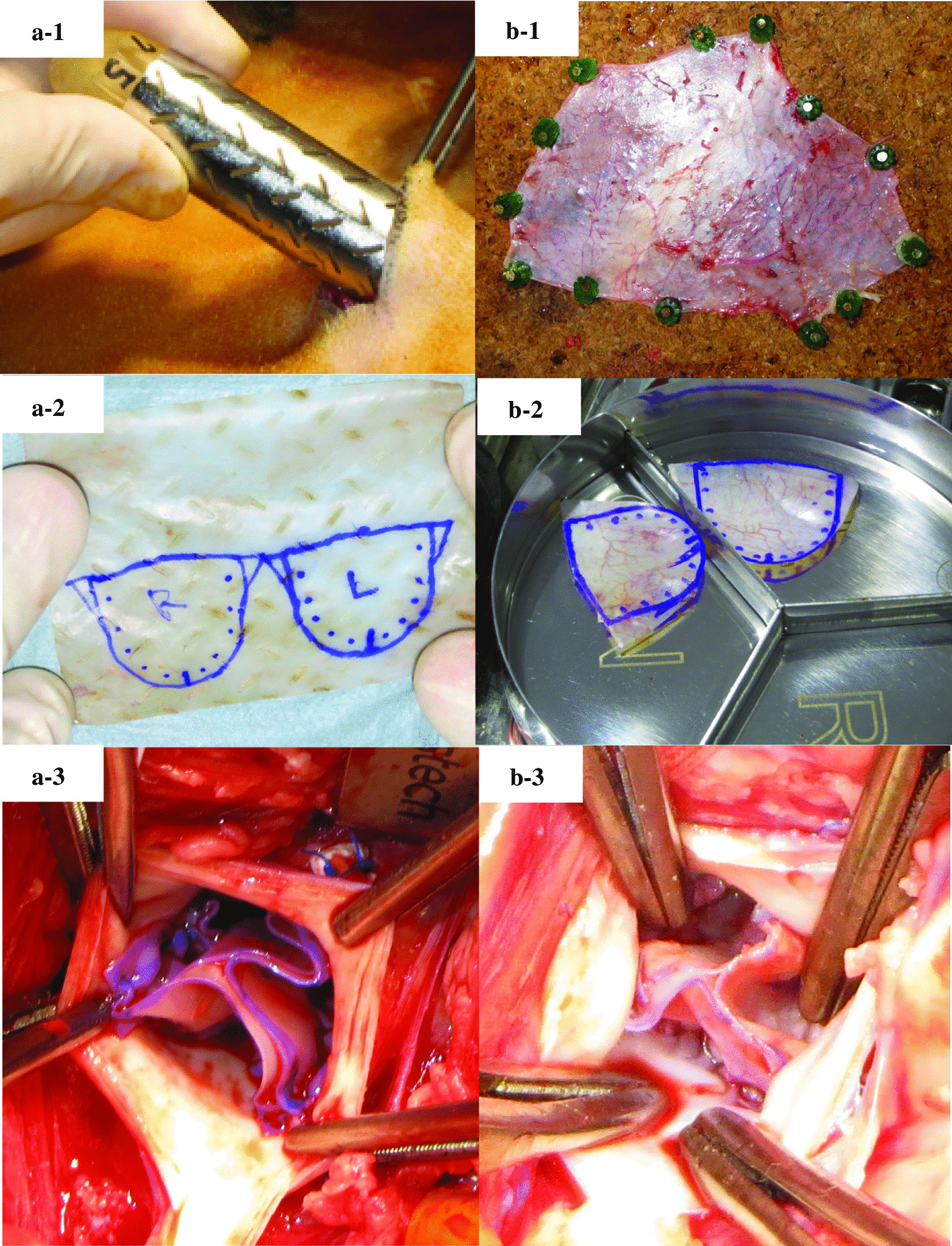
The mold was embedded for 2 months in a dorsal subcutaneous pouch in a goat model (**a-1**). After 2 months, the molds were encapsulated by the connective tissue membrane that formed thin flexible sheets (**a-2**). Three leaflets of biosheets were sutured with the annulus (**a-3**) The autologous pericardium was harvested (**b-1**). The autologous pericardium was cut to a leaflet shape (**b-2**). Three leaflets of the autologous pericardium were sutured with the annulus (**b-3**)

Molds with a herringbone surface pattern were assembled by inserting a silicone tube into a stainless steel pipe and fixing both ends with caps so that the tube was centered. The gap constituting the tissue formation space between the silicone tube and the stainless steel pipe was 1 mm. The stainless steel pipe had herringbone-pattern slits. Biosheets were prepared by embedding molds in the goats’ dorsal subcutaneous spaces under general anesthesia (Fig. 1a-1). Two months later the molds were extracted and kept in a 70 % ethanol solution. After two or three days, the tubular tissues formed in the molds were removed and dried indoors overnight. Then biosheets (5 × 7 cm) were obtained by cutting the tubular tissues in the longitudinal direction and cutting excess tissue from the sheet. The biosheets were then kept in a 70 % ethanol solution again for at least a week until AVRec. The biosheets were washed with physiological saline for 10 min just before implantation. In this study, the implanted biosheets originated from the same goats.

### Preparation of autologous pericardium

Adult goats underwent AVRec using autologous pericardium. The heart was exposed via a left thoracotomy at the third intercostal space. The autologous pericardium was harvested. The pericardium was excised with a size at least 5 × 7 cm (Fig. 1b-1). The excised pericardium was then treated with 0.6 % glutaraldehyde solution with saline for 10 min. The treated pericardium was rinsed thrice using a saline solution for 6 min.

### Aortic valve reconstruction

**Fig. 2:**
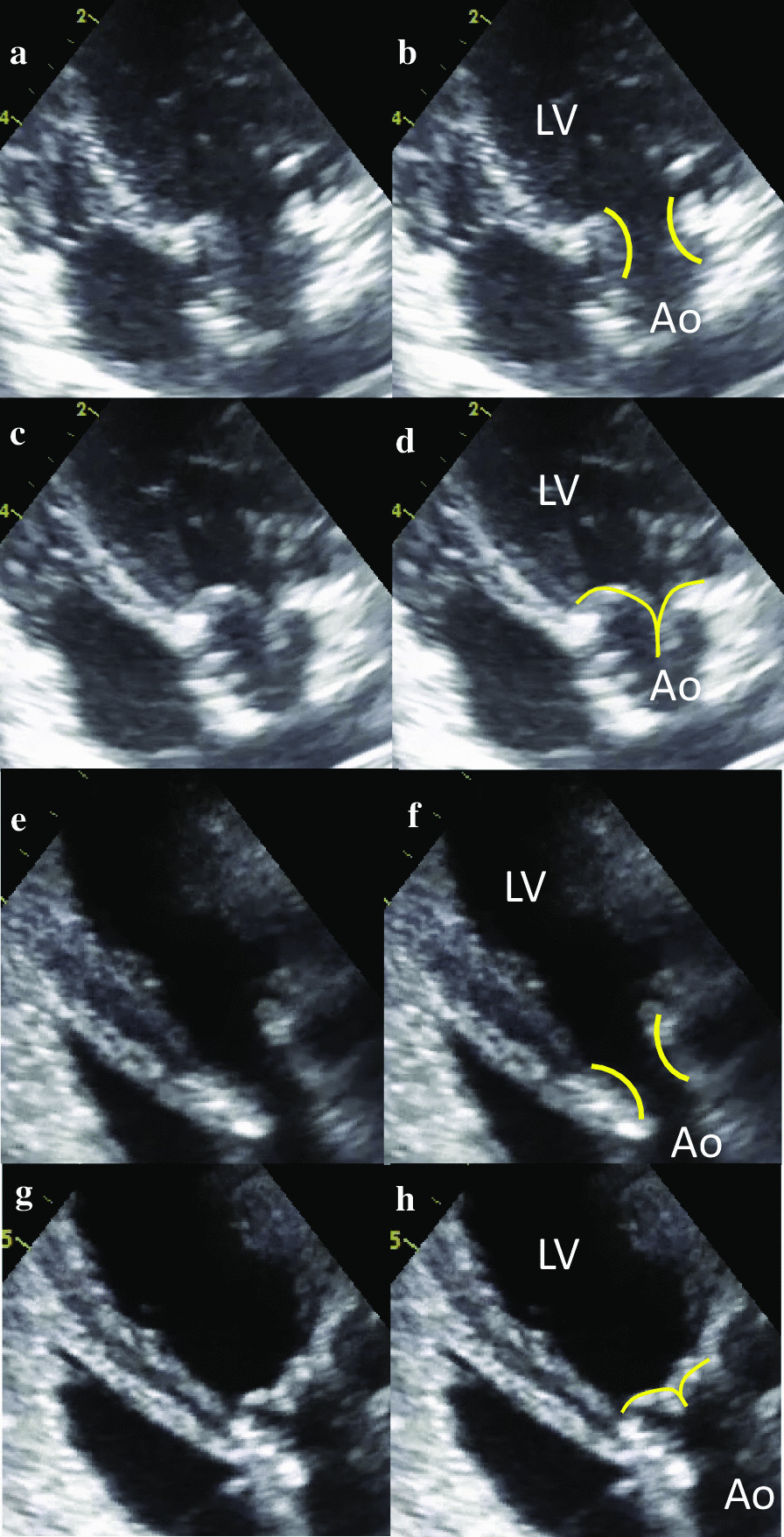
Movement of the leaflets in the aortic valve position by echocardiography 3 months after implantation. Both biosheet (systolic; **a**, **b** and diastolic; **c**, **d**) and autologous pericardium (systolic; **e**, **f** and diastolic; **g**, **h**) valve leaflets were smoothly opening and closing. Yellow lines show leaflets. The visible thrombus and cuspal tears were not detected. LV left ventricle, Ao aorta

**Fig. 3:**
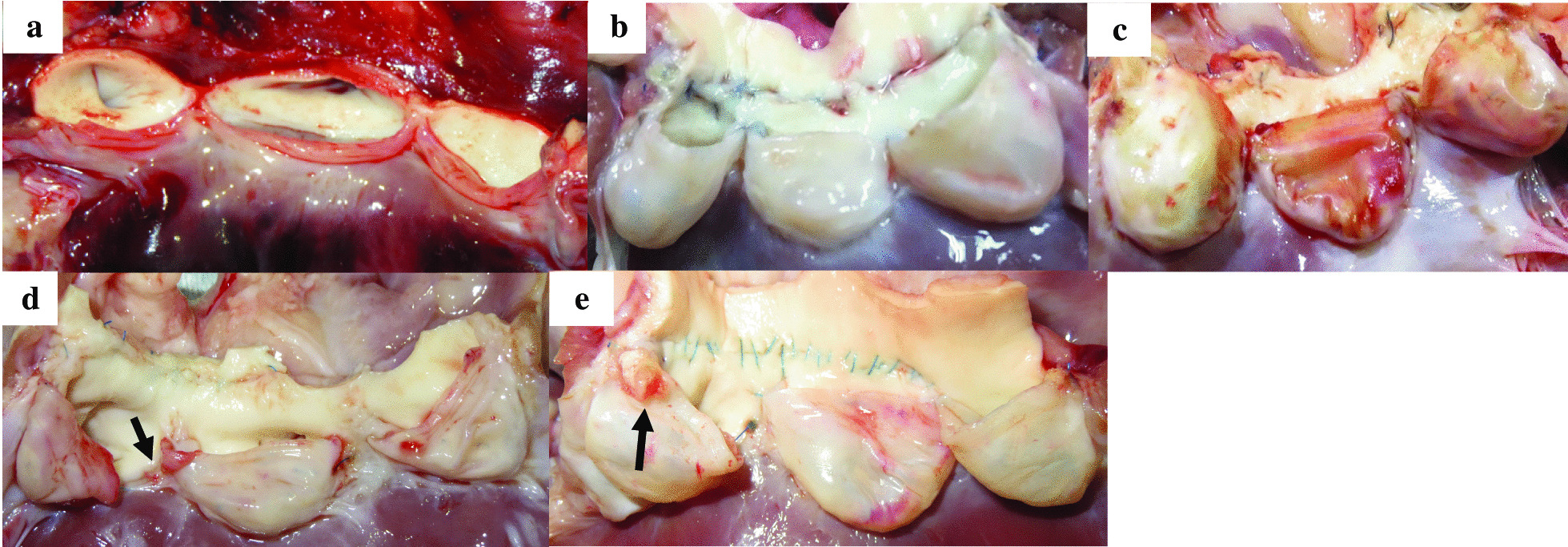
Photograph of the native aortic valve leaflets (**a**). Photograph of the harvested valve leaflets of biosheets (**b**) and of the autologous pericardium (**c**). Neither the biosheets nor the autologous pericardium showed thrombus formation on any of the leaflet surfaces. One of biosheet valves had a commissural cut (arrow) (**d**). One of biosheet valves from another goat had a cusp with a small grayish white protrusion (arrow) (**e**)

Cardiopulmonary bypass (CPB) was initiated following heparin administration (400 U/kg). The aortic arch was cannulated with an 18 Fr arterial cannula, and a 25 − 23 Fr two-stage venous drainage cannula was placed into the right atrium. The ascending aorta was cross-clamped, and then cardiac arrest was induced with cardioplegia. The ascending aorta was opened using a transverse aortotomy. After resection of the native aortic valve cusps, the distance between each commissure was measured using the Ozaki sizer. Both the biosheet and the autologous pericardium were cut to a leaflet shape using the Ozaki template corresponding to the measured size (Fig. 1a-<link rid="fig2”>2</link>, 1b-<link rid="fig2”>2</link>). The annular margins of the leaflets were attached to each annulus with 4 − 0 monofilament sutures (Fig. 1a-<link rid="fig3”>3</link>, 1b-<link rid="fig3”>3</link>). A 4 − 0 monofilament suture was used to close the aortotomy. The CPB was weaned off gradually and terminated. Epicardial and transesophageal echocardiography was performed. Finally, the chest wall was closed. We did not use anticoagulants or antiplatelet drugs after the surgery.

### Echocardiographic analysis

Epicardial echocardiography was performed at the end of the AVRec operation and at the time of sacrifice (3 months after AVRec). The left ventricular-aortic pressure gradient (LV-Ao PG) was measured, and aortic valve motion and aortic regurgitation (AR) were assessed. The semi-quantitative evaluation of AR was based on the ratio of the AR jet flow to the diameter of the left ventricular outflow tract (< 25 % = mild, 25–64 % = moderate, ≥ 65 % = severe). Echocardiographic studies were performed by the authors (surgeons). Those examinations were not blinded. After all the echocardiography data were collected, heparin (100 U/kg) was administered and the animal was euthanized via injection of pentobarbital sodium (120 mg/kg) and KCL (2 mEq/kg). The heart tissue was then harvested.

### Histological evaluation

The aortic biosheet valve extracted specimens were fixed with 10 % formalin, embedded in paraffin, sliced into longitudinal sections, and finally stained with hematoxylin–eosin (H&E), Elastica-van Giessen, Masson’s trichrome (collagen stain), and von Kossa (calcium stain). In addition, a few sections of the biosheet were also stained for α-smooth muscle actin (α-SMA) using immunohistochemical techniques; the protein was detected using monoclonal antibodies (Abcam, Cambridge, UK). The histological analyses were not blinded. Only one pathologist performed the pathological evaluation.

## Results

All the goats survived for three months after AVRec treatment with biosheets (n = 4) and glutaraldehyde-fixed autologous pericardium (n = 4).

### Echocardiographic analysis

The average of LV-Ao PG at AVRec operation and after 3 months was 10.8 ± 7.5 mmHg and 14.7 ± 9.8 mmHg in the biosheet group and 8.2 ± 5.8 mmHg and 15.6 ± 13.2 mmHg in the autologous pericardium group. Leaflet movement of all implanted valves were smooth at AVRec and after 3 months (Fig. 2). No aortic regurgitation was found immediately after AVRec in any case. However, three months later, there were some cases that developed AR in both groups (mild: n = 1, moderate: n = 1 in the biosheet group, mild n = 2 in the autologous pericardium group).

### Macroscopic findings

Biosheet group.

Upon removal three months after implantation, there was little change in size or shape in any of the biosheet leaflets. The harvested biosheets became transparent, similar to native aortic valve leaflets (Fig. 3a), and no thrombus was observed on their surfaces (Fig. 3b). A slight dissection at the commissure was observed in one biosheet leaflet (Fig. 3d), and another one had a cusp with a small grayish-white protrusion (Fig. 3e).

Autologous pericardium group.

Upon removal three months after implantation, the autologous pericardium leaflets had turned light brown, but there was minimal change in size or shape in all cases. The macroscopic appearances of the valve leaflets were similar to that of the native heart valve ones. No thrombus was observed on any luminal surfaces of the valves (Fig. 3c). Commissural cuts were not observed.

### Histological examination

**Fig. 4:**
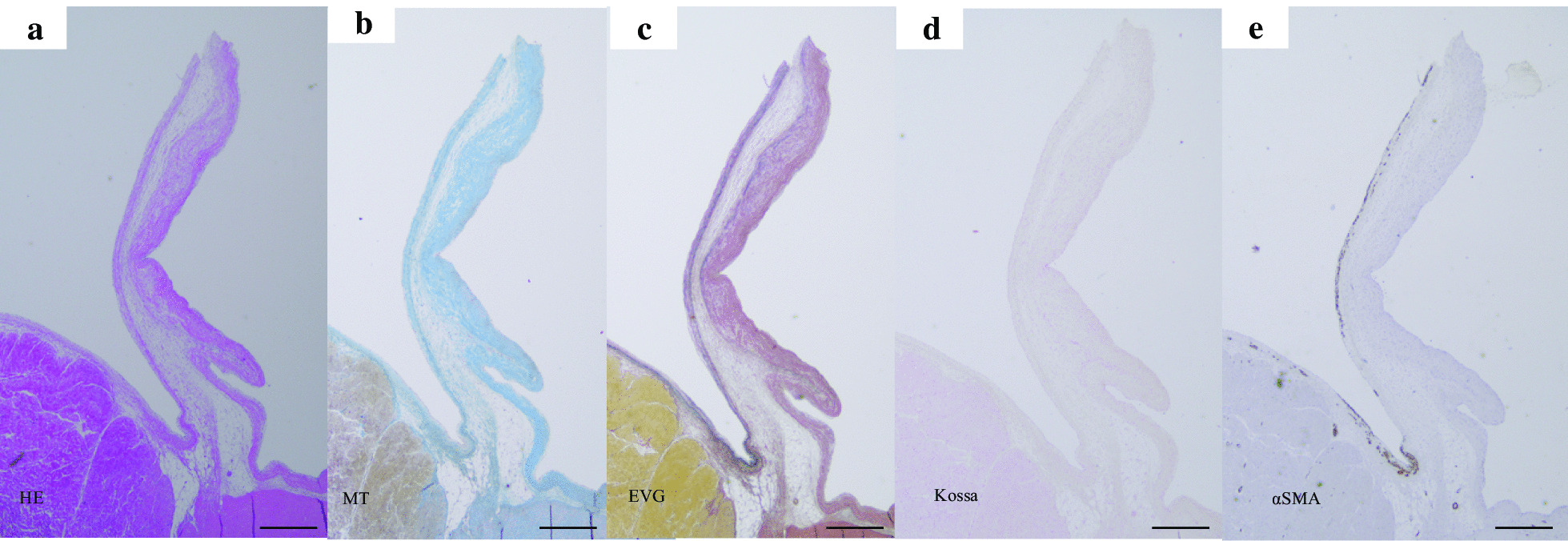
The longitudinal cross sections of native aortic valve leaflets (**a**-**e**). These were stained with hematoxylin–eosin (HE) (**a**), Masson’s trichrome (MT; collagen stain) (**b**), Elastica-van Giessen (EVG) (**c**), von Kossa (calcium stain) (**d**), and α-smooth muscle actin (α-SMA) (**e**). Scale bar equals 500µmm

The native aortic valve of a goat shows a thick collagen layer on the aortic side and an elastic fiber layer on the ventricular side both surrounded by endothelial cells with a smooth surface (Fig. 4).

**Fig. 5:**
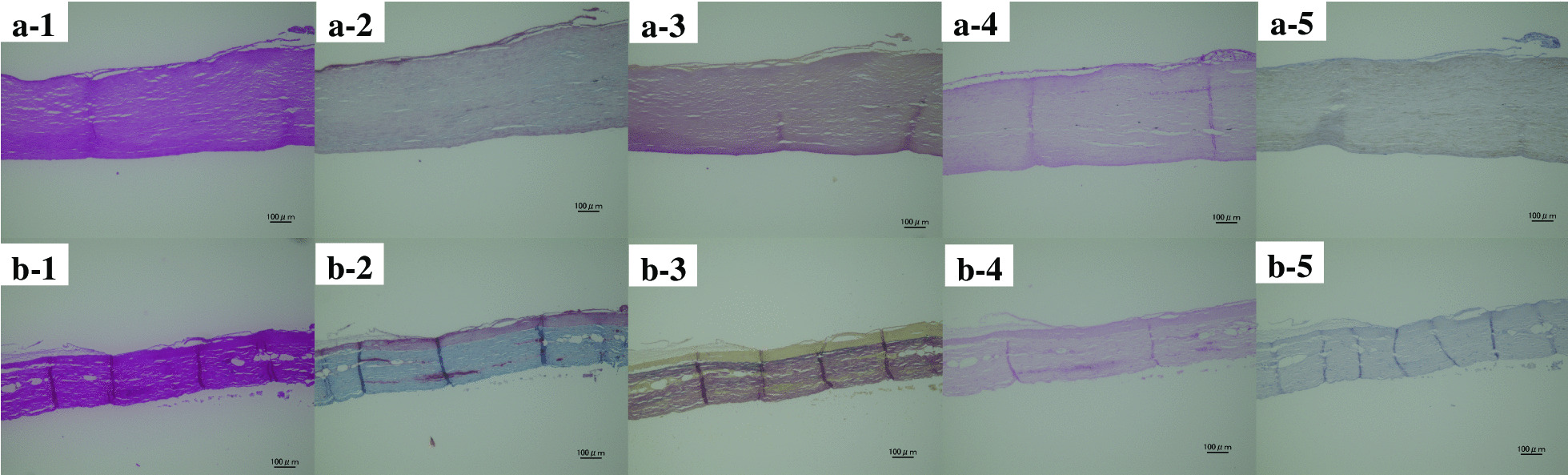
The longitudinal cross sections of biosheet (**a1-5**) and autologous pericardium (**b1-5**) were obtained before implanted. These were stained with hematoxylin–eosin (HE) (**a-1**, **b-1**), Masson’s trichrome (MT; collagen stain) (**a-2**, **b-2**), Elastica-van Giessen (EVG) (**a-3**, **b-3**), von Kossa (calcium stain) (**a-4**, **b-4**), and α-smooth muscle actin (α-SMA) (**a-5**, **b-5**). Scale bar equals 100 µmm

The collagen bundles of autologous pericardium leaflets were intermingled, but those of the biosheet were homogeneous before implanted. There were no a-SMA positive cells either in the biosheet or in the autologous pericardium (Fig. 5).

**Fig. 6:**
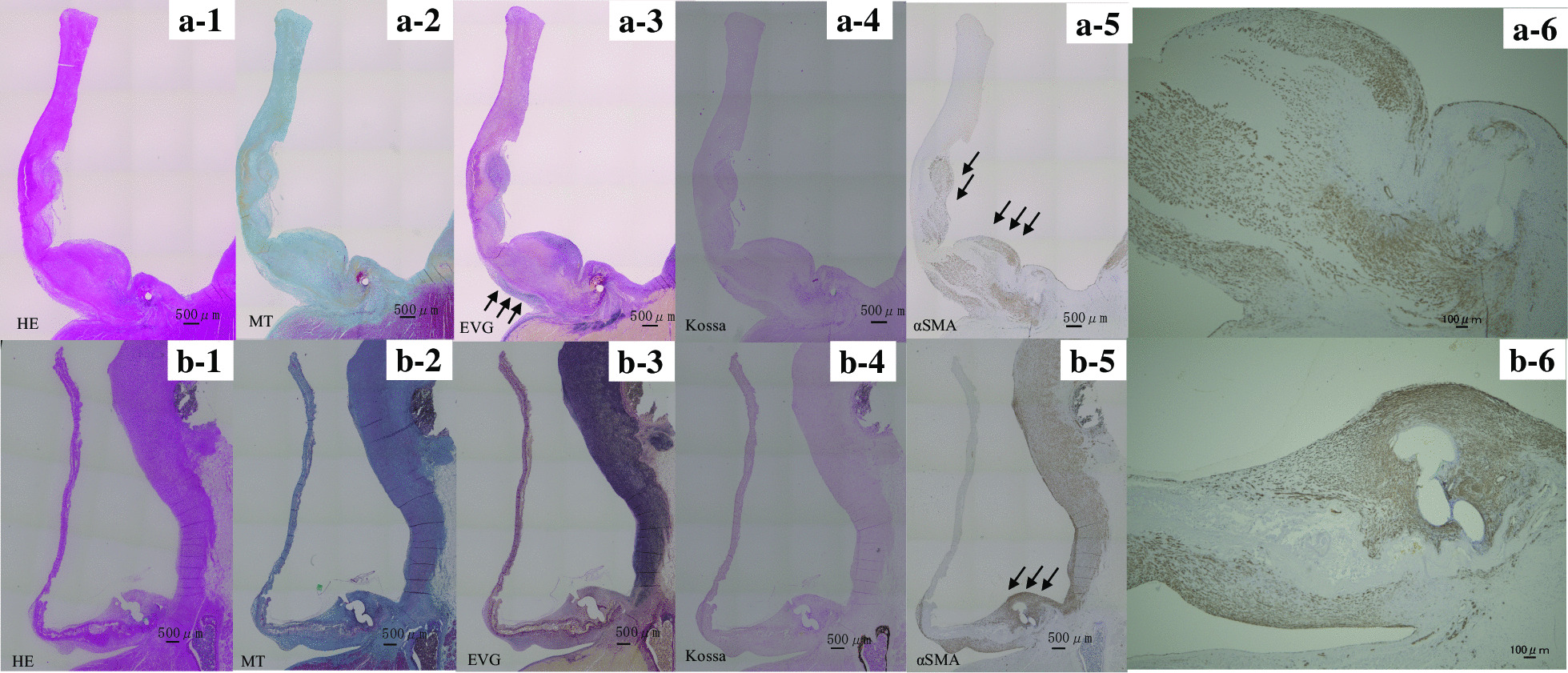
The longitudinal cross sections of valve leaflets of biosheets (**a1-6**) and of the autologous pericardium (**b1-6**) obtained 3 months after implantation and stained with hematoxylin–eosin (HE) (**a-1**, **b-1**), Masson’s trichrome (MT; collagen stain) (**a-2**, **b-2**), Elastica-van Giessen (EVG) (**a-3**, **b-3**), von Kossa (calcium stain) (**a-4**, **b-4**), and α-smooth muscle actin (α-SMA) (**a-5**, **b-5**). Scale bar equals 500µmm. Arrows in a-3 indicates elastic fibers. Arrows in **a-5** and **b-5** indicate α-SMA positive cells. **a-6** was high power field of a-5. b-6 was high power field of b-5. Scale bars on a-6 and b-6 equal 100 µmm

The surface was smooth all around, covered partially with a thin neointima in both materials 3 months after implantation (Fig. 6a-<link rid="fig1”>1</link>, b-<link rid="fig1”>1</link>). The base of the cusp was thicker than the other parts of leaflets in both groups. This might be because the base was the anastomosis site where tissue reaction occurs easily and cell infiltration first begins.

Both valves consisted of numerous collagen bundles, but the original pericardial fibrous tissue was almost unchanged since before implantation over the entire length of the valve (Fig. 6b-1,2). On the other hand, collagen bundles of the biosheet were disappearing near the aortic wall (Fig. 6a-1,2). Both materials had assimilated to an extent at the suture line of the aortic annulus as a tissue reaction, the boundary between the pericardial autograft leaflet and the reactive tissue was still clearly visible, whereas the boundary between the biosheet leaflet and the native annulus was unclear as it seemed that the new reactive tissue was replacing the attached biosheet. In the bases of cusps, the presence of myofibroblasts was confirmed in both sets of valve leaflets by α-SMA immunostaining; however, there were more myofibroblasts in the biosheet than in the pericardial autograft leaflets (Fig. <link rid="fig6”><link rid="fig6”>6</link></link>a-<link rid="fig5”>5</link>,<link rid="fig6”>6</link>, b-<link rid="fig5”>5</link>,<link rid="fig6”>6</link>). Besides, in biosheet valves elastic fibers that did not exist before had grown up to one-third of the way from the valve base on the ventricular side but no elastic fibers were found in pericardial valves (Fig. 6a-<link rid="fig3”>3</link>, b-<link rid="fig3”>3</link>). There were no calcifications observed in either the biosheet valves or the pericardial valves (Fig. 6a-<link rid="fig4”>4</link>, b-<link rid="fig4”>4</link>).

Bacterial debris and neutrophil infiltration in the small protrusion of the biosheet cusp was compatible with healing endocarditis.

## Discussion

Valvular dysfunction due to heart disease requires frequent reconstruction with the use of mechanical valves, biological valves, or autologous pericardium valves. Autologous pericardium is commonly used as a plastic material in intracardiac structures. For AVRec using the Ozaki procedure, autologous pericardium is used after glutaraldehyde fixation. This has produced excellent clinical results, but glutaraldehyde fixation limits growth adaptability. For young patients, there are concerns regarding the insufficient amount of the pericardium in reoperation cases, the decreased coaptation due to growth, and the undetermined durability of the pericardium [9].

Some papers have reported that biosheets based on iBTA can be reconstructed in a manner analogous to that of native tissue, for example, as cornea, trachea (biosheet), and blood vessels (biotube) [7, 8]. The technology based on iBTA has also been applied to heart valves (biovalve and stent-biovalve) [10, 11] and stent grafts (bio stent graft) [12]. The papers that reported on this technology indicated the possibility of tissue regeneration, self-repair, and growth adaptability.

To our knowledge, this is the first time that tissue-engineered valve leaflets have been successfully implanted as aortic valves using goats as a large animal. It was amazing that all goats survived for three months after implanting biosheets without requiring reinforcement treatment.

Biosheets were more thoroughly assimilated into the aortic root than the autologous pericardium. On histology, a greater number of smooth muscle actin-positive cells (myofibroblasts) had infiltrated into biosheet leaflets than in the autologous pericardium. This indicates the biosheet had more neointima and myofibroblasts than the autologous pericardium. We think we should analyze these histopathological changes in more detail in the future, comparing existing results with longer survival models.

On the other hand, it was unfortunate that one biosheet leaflet had a slight cut at the suture with the aortic annulus. The Biosheet was fairly durable and easy to handle in use so that technically there was no difference between AVRec with the biosheet and with the autologous pericardium. AR might have been caused by this cut, not by any technical difficulty with the biosheet. It is also possible that the biosheet used had insufficient strength, or it may not have had long-term durability due to an unidentified defect. Therefore, in our other research, we investigated the physical properties of biosheets. A biosheet is formed in a mold, and thus, depends on the pattern of openings in the mold which function as cell entry ports. In the study of the tensile strength of biosheets according to the mold pattern (alternating “herringbone” pattern or parallel pattern) [13], we compared the tissue stress of the circumferential direction with that of the longitudinal direction. The biosheets with an alternating herringbone pattern had low anisotropy. Therefore, the selection of appropriate biosheets as candidate materials for the substitution of the aortic leaflets should be taken into consideration in our future work. Artificial heart valves have also been developed using polymeric materials such as Poly(Styrene-block-IsoButylene-block-Styrene (SIBS). In a typical SIBS, the ultimate tensile strength is 5–30 (MPa) [14, 15], and if the biosheet is 1.0 mm, it is 10 MPa or more. We decided to make the thickness of the biosheet 1 mm in reference to the previous experiment [13]. We thought that a 1 mm thick valve membrane at the aortic valve position would be sufficient. It should be noted that this thickness of 1 mm was the thickness right after the biosheet was taken out of the mold and that it thinned to about 0.5 mm just before AVRec after being treated with 70 % ethanol. The thickness of the autologous pericardium valve just before implantation was about 0.25 mm and the thickness of the native aortic valve was about 0.4 mm. We would like to carry out future research in determining the optimal thickness. The thickness of the biosheet can be tailored by adjusting the size of the gap of the mold. It would even be possible to make the biosheet with a different thickness at the tip and base. Unfortunately, one of the biosheet model subjects developed infectious endocarditis. However, chronic animal models may develop infectious endocarditis as one of the complications of cardiac surgery; we believe the infection was not caused by the biosheet.

In the technology based on iBTA, the recipient’s body is used as a bioreactor. This technique has several advantages. For example, the tissue prostheses can be easily and safely fabricated in a wide range of shapes and sizes to suit an individual recipient by changing the mold design. Most importantly, this technique does not require complex in vitro cell management procedures or exceptionally clean laboratory facilities, which are extremely expensive and time consuming. Moreover, because the biosheets are completely autologous, they are expected to have little calcification with long-term implantation and to possess better growth potential when compared with mechanical valves or biological valves. Consequently, biosheets might be an ideal material for AVRec. One of the most important features of iBTA- induced tissue is its ability to regenerate.

The current study demonstrated a few experiments using the biosheet. Its timeframe was short. We gave priority to tissue observation this time and separated the tissue at 3 months because another article regarding iBTA showed that histopathological change occurred at 3 months [6]. We are now increasing the number of experiments after careful selection of suitable biosheets for implantation. Further investigations on long-term histological features and durability are ongoing considering the possibility of future clinical use for humans.

### Limitations of the study

Although this study included large animal models, the number of animals used might not be enough to generalize the results. Also, as one of the biosheet models required cutting of tissues we used thin biosheets, which may have affected the strength of the biosheet. In the future, the quality of biosheets must be confirmed before implantation.

To improve the selection of biosheets with an appropriate thickness, we developed a system that can measure the total thickness of this material using optical coherence tomography (OCT, IVS-2000, Santec, Aichi, Japan). We expect the surgical environment will improve, including the cleaning treatment of biosheets to prevent infection. Checking the number of bacteria before AVRec surgery and using clean biosheets can help avoid complications. Finally, our limitations must be considered while applying the results of this study for clinical use.

## Conclusions

For the first time, biosheets were used for large animal AVRec without glutaraldehyde fixation. In this limited preliminary study, it was found that biosheets could function as leaflets in the aortic position and may have the ability to assimilate into native valves. Hence, this study can be considered as a preliminary study on the use of biosheets in AVRec.

## Data Availability

The datasets that support the findings of this study are available from the corresponding author on reasonable request.

## Abbreviations

AR: Aortic regurgitation
AVRec: Aortic valve reconstruction
iBTA: In-body tissue architecture
CPB: Cardiopulmonary bypass
LV-Ao PG: Left ventricular-aortic pressure gradient
H&E: Hematoxylin–eosin
αSMA: α-smooth muscle actin
